# Supplementary material for: The impact of core self-evaluation on school adaptation of high school students after their return to school during the COVID-19 pandemic: the parallel mediation of positive and negative coping styles
Source: PeerJ. 2023 Oct 31;11:e15871. doi: 10.7717/peerj.15871 (PMC10624169; doi:10.7717/peerj.15871)
Supplement: Supplemental Information 2 [file peerj-11-15871-s002.docx]

Variable assignment table

| Study variables | Assignment |
| --- | --- |
| Gender | 1=Male，2=Female |
| Core self-evaluation | 1=completely inconsistent, 2=relatively inconsistent, 3=uncertain, 4=relatively consistent, 5=completely consistent |
| Coping style | 0=not taken, 1=occasionally taken, 2=sometimes taken, 3=frequently taken |
| School adaptation | 1=completely inconsistent, 2=relatively inconsistent, 3=uncertain, 4=relatively consistent, 5=completely consistent |
